# Absence of Hepatitis E Virus (HEV) Circulation in the Most Widespread Wild Croatian Canine Species, the Red Fox (*Vulpes vulpes*) and Jackal (*Canis aureus moreoticus*)

**DOI:** 10.3390/microorganisms11040834

**Published:** 2023-03-24

**Authors:** Jelena Prpić, Ana Kunić, Tomislav Keros, Ivana Lojkić, Dragan Brnić, Lorena Jemeršić

**Affiliations:** Croatian Veterinary Institute, Savska cesta 143, 10000 Zagreb, Croatia

**Keywords:** hepatitis E virus, red fox, jackal, wild animals, zoonosis, seroprevalence, viral persistence, Croatia

## Abstract

Hepatitis E virus (HEV) can infect a wide range of domestic and wild animals, and the identification of new host species is reported successively worldwide. Nevertheless, its zoonotic potential and natural transmission, especially in wildlife remains unclear, primarily due to the discrete nature of HEV infections. Since the red fox (*Vulpus vulpus*) is the most widespread carnivore worldwide, and has been recognized as a potential HEV reservoir, its role as a potent host species is of increasing interest. Another wild canine species, the jackal （*Canis aureus moreoticus*）, is becoming more important within the same habitat as that of the red fox since its number and geographical distribution have been rapidly growing. Therefore, we have chosen these wild species to determine their potential role in the epidemiology and persistence of HEV in the wilderness. The main reason for this is the finding of HEV and a rather high HEV seroprevalence in wild boars sharing the same ecological niche as the wild canine species, as well as the risk of the spread of HEV through red foxes into the outskirts of cities, where possible indirect and even direct contact with people are not excluded. Therefore, our study aimed to investigate the possibility of natural HEV infection of free-living wild canines, by testing samples for the presence of HEV RNA and anti-HEV antibodies to gain better epidemiological knowledge of the disease. For this purpose, 692 red fox and 171 jackal muscle extracts and feces samples were tested. Neither HEV RNA nor anti-HEV antibodies were detected. Although HEV circulation was not detected in the tested samples, to our knowledge, these are the first results that include jackals as a growing and important omnivore wildlife species for the presence of HEV infection in Europe.

## 1. Introduction

The public health significance of hepatitis E is enormous. According to the World Health Organization (WHO), there are an estimated 20 million hepatitis E virus (HEV) infections worldwide every year, leading to an estimated 3.3 million symptomatic cases of hepatitis E [1]. The WHO estimates that hepatitis E caused approximately 44,000 deaths in 2015, which represents 3.3% of the mortality due to viral hepatitis. Hepatitis E virus was first recognized during an epidemic of hepatitis that occurred in the Kashmir Valley in 1978. The epidemic involved an estimated 52,000 cases of icteric hepatitis with 1700 deaths [2]. For years, HEV was believed to be endemic in places with poor biosecurity and hygiene measures and was, therefore, considered travel-related. Nowadays, hepatitis E represents an emerging zoonotic infection in many European countries [2]. It is estimated that 5–15% of all acute hepatitis infections of unknown origin in Europe are caused by HEV [1]. Hepatitis E is an enterically transmitted infection caused by a small (27–34 nm) non-enveloped, single-stranded RNA virus [3]. The hepatitis E virus linear genome of positive polarity with a length of approximately 7.2 kb consists of a short 5′ untranslated region (UTR), three partially overlapping open reading frames (ORFs), and a 3′ UTR [4,5]. The 5′ UTR contains a 7-methylguanosine cap (7 mG), which is essential for the initiation of HEV replication and infectivity, and the 3′ UTR is polyadenylated (polyA). ORF1 encodes a nonstructural polyprotein with multiple potentially functional domains: methyltransferase (Met), Y domain, papain-like cysteine protease (PCP), hypervariable region (HVR), X domain, helicase (Hel), and RNA-dependent RNA polymerase (RdRp). It is still debatable whether the ORF1 polyprotein undergoes processing into individual functional proteins. The ORF2 encodes the viral capsid protein, which contains the S, M, and P domains. The ORF3 overlaps partially with ORF2 and encodes a small phosphoprotein that is necessary for viral release [6]. According to the classification released in 2021 (International Committee on the Taxonomy of Viruses, ICTV) [7], HEV is classified in the genus *Orthohepevirus* of the family *Hepeviridae*, and it shows a relatively strict host specificity [8]. The human-associated genotypes, which are the leading cause of acute hepatitis worldwide, as well as zoonotic genotypes originating from domestic animals and wild-living mammal species (pig, wild boar, rabbit, deer, mongoose, and camel species), are grouped into the species *Orthohepevirus A*, which includes a total of eight genotypes (HEV1–8). *Orthohepevirus B* contains the avian hepatitis E virus species causing the “splenomegaly syndrome” as well as the “big liver and spleen disease” in poultry, whereas *Orthohepevirus C* viruses were isolated from rodents (rats, mice, voles, and shrew) and carnivores (such as ferrets, mink, and foxes). HEV from bats is classed in the species *Orthohepevirus D*. Finally, fish-related HEV belongs to the genus *Piscihepevirus* [8,9,10,11]. Members of the genus *Orthohepevirus* infect a wide range of animals, although the exact host range remains obscure, primarily due to the discrete nature of HEV infections. HEV often presents undetectable pathology in infected organisms. Usually, the viral load remains low, and the viral shedding is prolonged or chronic [12]. Chronic HEV infection is a rare event and occurs mainly in immunosuppressed populations, especially in solid organ transplant recipients [12]. Numerous monitoring studies have been performed in Europe in the past in order to determine HEV circulation in the animal population. So far, among the Hepatitis E viruses, genotypes of the *Orthohepevirus A* species represent the most common cause of acute hepatitis in humans worldwide [10]. Domestic pigs and wild boars are considered the main reservoirs of the virus and a potential source of zoonotic transmissions based on serological and molecular results [13]. Hepatitis E infection is mainly transmitted through the consumption of contaminated food or water [14]. Direct contact transmission has been demonstrated in domestic pigs [15,16]. There are reports available that associate hepatitis E with the consumption of raw or undercooked food products of pigs, wild boar, deer, or contaminated shellfish [17,18,19,20]. In comparison to the general population, a statistically higher seroprevalence is found in pig farmers and veterinarians [21]. This suggests that contact exposure to domestic pigs may also be a risk factor.

The first HEV strain isolated from carnivore species was obtained from household pet ferrets (*Mustela putorius*) in the Netherlands [22]. The isolation of an HEV strain from farmed American minks (*Neovison vison*) in Denmark [23] has also been described. Foxes can be considered as natural reservoir hosts of several pathogens and as effective vectors of zoonotic diseases that pose an important risk for domestic animals and humans as well [24,25,26,27]. So far, there are only limited serological or molecular data available for HEV prevalence in foxes. HEV strains have been reported only in red foxes (*Vulpes vulpes*) from the Netherlands [28], Germany [29], and Hungary [30]. Sequencing of detected HEV strains from foxes showed high nucleotide identity with a rodent-related HEV group, which was the exclusively known group within the *Orthohepevirus C* species. These findings support “the dietary origin” of unclassified HEV-like strains described from various predator species, since most of these variants were detected in fecal samples of foxes, considering that these wild carnivores generally consume rodents [30,31,32]. Multiple detections of the rodent-related variants from different geographical locations may suggest that these carnivores are reservoirs of the virus [33]. There is a lack of available data regarding HEV infection in jackals worldwide. Only one available report from China shows the absence of HEV infection in jackals [34]. However, since the number as well as the geographical distribution of this wild canine species is growing, and as omnivores, predators, and scavengers, the possibility of the jackal as a potential reservoir of HEV cannot be excluded.

In Croatia, the first samples of animal origin were tested for the presence of HEV RNA in 2007 [35], and a comprehensive survey based on viral RNA detection in domestic and wild animals and mollusks with the aim of exploring the possible role of wild animals in the spread of the zoonotic HEV genotypes was carried out in 2009 [36]. HEV RNA was detected in domestic pigs and wild boars, but it was not confirmed in any other domestic or wild animals. A study regarding the HEV seroprevalence in domestic pigs and wild boars in Croatia was carried out in 2016 [37,38]. The reported HEV seroprevalence found in domestic pigs and wild boars in Croatia corresponded to results reported in other European countries [39,40,41,42,43]. The main trigger for HEV testing in these two wild canine species was the fact that red foxes and jackals share the same habitat as free-living wild boars, which have been proven to be reservoirs of HEV in Croatia. Additionally, the possibility of transboundary HEV transmission and spread through wild boar migration has been reported previously [37]. Additionally, the finding of a yellow-necked mouse (*Apodemus flavicollis*) naturally infected with HEV strains grouped into genotype 3 of *Orthohepevirus A* species [44] in Croatia was one of the reasons for HEV testing in these two wild canine species, since both species feed mostly on small rodents.

## 2. Materials and Methods

### 2.1. Sample Collection and Preparation

**Figure 1:**
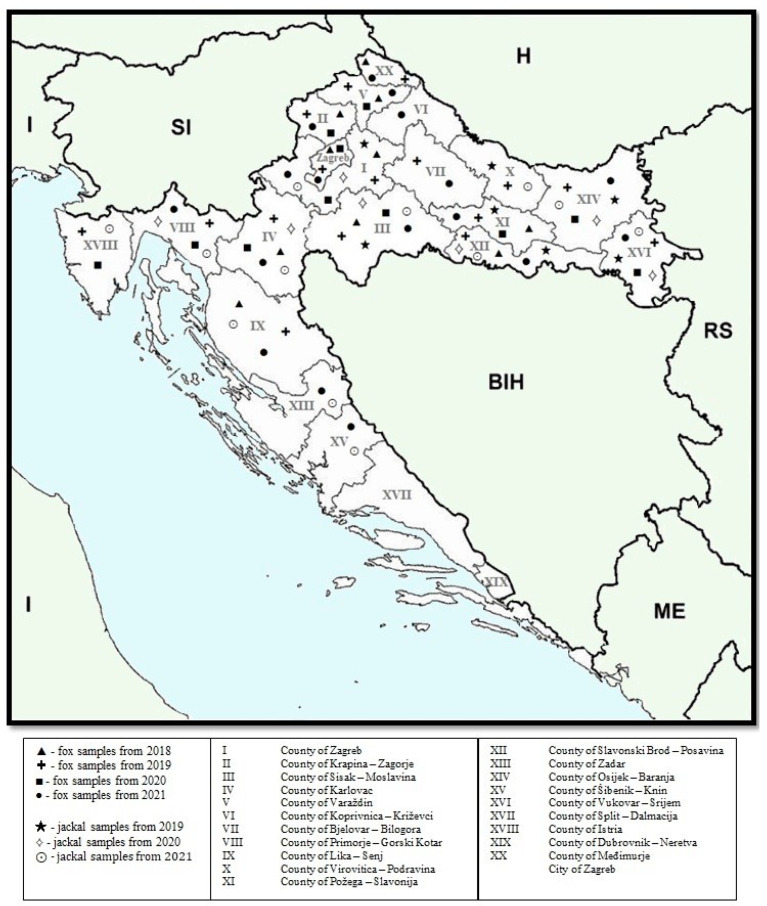
Map of Croatia indicating the regions where the red fox and jackal samples were collected. Countries with international borders to Croatia are Bosnia and Herzegovina (BIH), Hungary (H), Montenegro (ME), Serbia (RS), and Slovenia (SI); Croatia shares a maritime border with Italy (I) in the Adriatic Sea.

Muscle extracts and feces samples of red foxes (*Vulpes vulpes*) and European jackals (*Canis aureus moreoticus*) were collected from 2018 to 2021 according to an ongoing national rabies annual monitoring program prescribed by the Croatian Ministry of Agriculture, Veterinary and Food Safety Directorate, since no HEV monitoring program has been established in Croatia. Samples were randomly chosen, taking into account the sample quality and geographical origin (Figure 1). Muscle samples were collected immediately after the animal’s death.

**Figure 2:**
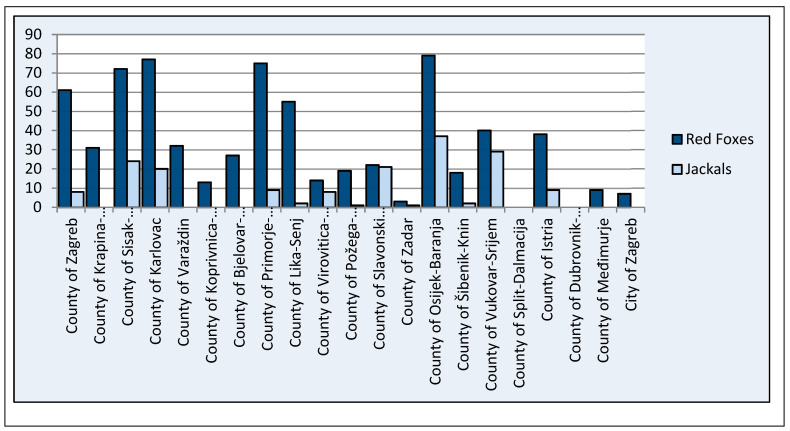
Number of red fox and jackal samples tested per county.

In total, 692 red foxes (76 feces samples in 2018, 185 in 2019, and 48 in 2020 and 383 muscle extract samples in 2021) and 171 jackals (20 feces samples in 2019, 37 in 2020, and 71 in 2021 and 43 muscle extract samples in 2021) were tested for HEV presence (Figure 2, Table 1). Muscle extract samples were tested for presence of anti-HEV antibodies and for the presence of HEV RNA, whereas feces samples were tested for the presence of HEV RNA.

A segment of musculus femoralis of approximately 5 × 7 cm was taken from fox/jackal carcasses by trained pathology technicians at the Croatian Veterinary Institute Pathology laboratories. The muscles were placed in polypropylene containers (security screw cap containers, 120 mL, DeltaLab), snap-frozen, and stored at −80 °C for four days and then kept at 4 °C for 3–5 days. From each sampled muscle piece, approximately 200–500 µL of the muscle extract was collected. Muscle extracts were centrifuged for 10 min at 220× *g* and then stored at −20 °C until use. Before being used as the starting material for RNA extraction and ELISA, all samples were heat-treated at 56 °C for 30 min and centrifuged for 10 min at 220× *g*. Fecal samples were resuspended in phosphate-buffered saline (PBS, pH 7.4) to obtain 20% *w/v* fecal suspensions, which were then vortexed for 30 s and centrifuged for 3 min at 14,000× *g*. Supernatants were further used as the starting material for RNA extraction. The exogenous internal positive control (IPC) RNA Xeno™ RNA Control (ThermoFisher Scientific, Waltham, MA, USA) was added to each sample (2 µL) to monitor the appearance of potential PCR inhibitors.

### 2.2. RNA Extraction and HEV Detection through Real-Time RT-PCR

Viral RNA was extracted from 200 μL of supernatant of prepared fecal/m. femoralis samples using a MagMAX Core or MagMAX Pathogen RNA/DNA Kit (Thermo Fisher Scientific, Waltham, MA, USA) on a KingFisherTM Flex purification system (Thermo Fisher Scientific, Waltham, MA, USA) according to the manufacturer’s instructions. RNA extracts were stored at −80 °C until use. To identify HEV RNA carriers, a one-step real-time RT-PCR protocol [45] for detecting highly conserved fragments within ORF3 was carried out. This rapid and sensitive broad-range real-time RT-PCR assay is used for the detection of HEV genotypes of *Orthohepevirus A* species. In brief, the amplification was carried out with a commercially available kit (4 × 1 Step RT qPCR Probe kit, highQu, Kraichtal, Germany), primers JVHEVF (5′-GGTGGTTTCTGGGGTGAC-3′) and JVHEVR (5′-AGGG-GTTGGTTGGATGAA-3′), and probe JVHEVP (5′-6-FAM-TGATTCTCAGCCCTTCGC-3′BHQ) according to the producer’s instructions. The amplification was carried out in a CFX Touch System (Bio-Rad, Hercules, California, USA) according to an established protocol (reverse transcription for 5 min at 50 °C, RT inactivation/PCR activation for 3 min at 95 °C, 40 cycles of 15 s denaturation at 95 °C, and 30 s annealing/elongation at 58 °C). Positive control sequences of previously detected fragments of HEV RNA genotype 3 were used (derived from positive swine sera submitted to GenBank under accession no. KT583116, grouped into HEV3c subtype, with Ct-value 29). Negative controls were aliquots of ultrapure water. Standard precautions were taken to prevent PCR contamination including a closed system for PCR amplification/detection. Additionally, the preparation of primers, PCR mastermix, RNA extraction, and the final addition of RNA were carried out in separate laboratories.

### 2.3. Detection of Specific Anti-HEV Antibodies through Enzyme-Linked Immunosorbent Assay (ELISA)

In order to detect total anti-HEV antibodies in muscle extracts, a multispecies ELISA Kit (ID Screen^®^ Hepatitis E Indirect Multi-species ID-Vet, Grables, France) was used, following the manufacturer’s instructions. This indirect immunoassay is applicable for the detection of HEV antibodies in multiple mammal species. The microplate is read and optical density is recorded at 450 nm with an automatic ELISA processor, ETI-Max3000 (DiaSorin, Sallugia, Italy).

## 3. Results

A total of 692 red fox and 171 jackal muscle extract/feces samples were analyzed to evaluate the presence of HEV RNA. Despite the efficient RNA extraction (the result of IPC amplification reveals the general absence of PCR inhibitors in the tested samples), HEV RNA was not detected in analyzed samples. The exposure of these animal species to HEV was also assayed through detecting anti-HEV antibodies in muscle extract samples. None of the samples were positive for antibodies.

## 4. Discussion

Throughout history, wildlife has been a significant source of infectious diseases transmissible to domestic animals and humans [46]. Wildlife can be considered the major reservoir of various bacteria, viruses, and parasites, whereas fungi are of negligible importance [47]. Three quarters of all emerging infectious diseases of humans are zoonotic, 70% of which are of wildlife origin [46,48,49,50]. The current rapid ecological changes in the world have negative impacts on pathogenic organisms, their vectors, and hosts, which are equally capable of rapid change [51]. Viral diseases originating from wild animals are widely considered major threats to public health, and the transmission of such viral pathogens from wildlife to other domestic animals and humans remains an important scientific challenge hampered by pathogen detection limitations in wild species [52]. The domestication of animals, human population expansion and encroachment into wildlife habitats, reforestation and other habitat changes, pollution, and the hunting of wild animals are key anthropogenic activities driving viral disease emergence at the global scale and in most instances, these activities have contributed to wildlife population decline and extinction [53]. The movement of pathogens, vectors, and domestic animals, as well as humans, is another factor influencing the epidemiology of wildlife-related disease (viral, bacteria, and parasitic) outbreaks. Such movements are commonly encountered at the edges of protected areas due to the availability of rich resources, and they bring about interactions at the wild/domestic animals/humans interfaces, with conflicts and potential for pathogen spread at these interfaces. These are emerging threats to wildlife conservation goals and livestock and human health.

Since the first report of HEV genotypes belonging to the *Orthohepevirus C* species in Norway rats (*Rattus norvegicus*) [54], several new Rat HEV-like sequences have been described in other animal species, including carnivores and birds of prey [10,11]. All of these new strains were found in fecal samples, so it might be possible that the virus identified in predator animals is originally derived from prey rodent species.

In the federal state of Brandenburg, Germany, a comprehensive HEV surveillance study with a unique panel of fox transudate samples, which were collected over 20 years, was performed [29]. The detected high antibody prevalence of the virus demonstrated the presence of endemic HEV infections within a fox population in Germany, and the HEV genome sequences clustered into the *Orthohepevirus C* group. An HEV survey on red fox fecal samples performed in Hungary supported the “dietary origin” of unclassified HEV-like strains described from predators that usually feed on rodents, since their sequences displayed high similarity to common vole HEV derived from *Microtus arvalis* [30].

Nevertheless, our study showed that none of the tested red fox and jackal samples were positive for the presence of HEV genotypes belonging to the *Orthohepevirus A* species or anti-HEV antibodies despite the factors that determined the choice of animals (geographical habitat). The included samples of red foxes and jackals were from the area where wild boars were previously shown to be positive, from the area where a high presence of wild rodents is detected, and from the area where a wild mouse was previously found to be positive for HEV. One of the explanations could be that the primers and probe set used is not specific for HEV genotypes belonging to the *Orthohepevirus C* species. We used a real-time RT-PCR assay for the detection of HEV genotypes of the *Orthohepevirus A* species since the strains previously derived from Croatian wild boars, domestic pigs, wild mouse, and humans were genetically highly related [36,37,38,44]. Previous findings indicated that small rodents in Croatia could be an epidemiological ‘link’ in the HEV transmission to other wild and domestic animals, and even humans. Additionally, it can be presumed that the negative ELISA results are a consequence of the sample nature. We tested muscle extract samples, not sera or plasma samples as recommended by the manufacturer of the commercial ELISA kit used. Using muscle extract samples in order to detect specific antibodies has been reported to be successful for assessing the efficiency of the rabies eradication program in red foxes [55]. The detection of HEV antigen (Ag) has been suggested as a convenient and cost-efficient alternative to RT-PCR, since HEV Ag production may parallel that of HEV RNA. It was reported that the method of HEV Ag detection had good concordance with HEV RNA detection and could serve as a useful tool in the early diagnosis of infection [56,57]. However, the detection efficiency of HEV Ag greatly diminished when the HEV RNA level was low or the anti-HEV IgG level was high [58]. Additionally, due to the low sensitivity/specificity/accuracy of the Ag assay, RT-PCR was used for the identification of HEV RNA carriers in this study.

Since no HEV-associated clinical signs in foxes are known to date, it is assumed that this species may constitute a reservoir species for hepatitis E infection [59]. However, no information about the virulence of HEV in foxes and any possible impact on the morbidity and mortality of the fox population is available so far [60]. Foxes are the most widespread predators throughout the world, and have been recognized as potential reservoirs of zoonotic pathogens including trematodes, cestodes, and nematodes [61] as well as *Babesia* spp. and *Theileria* spp. [62].

Since urbanization is a driving force for the emergence of zoonotic diseases and a major risk factor for the transmission of such agents to humans, the tendency of foxes to establish populations in suburban and urban areas should be kept in mind [60]. Alveolar echinococcosis caused by *Echinococcus multilocularis*, which displays transmission routes similar to HEV including fecal shedding and subsequent ingestion of the pathogen, is an excellent example for the dispersal of a fox-derived zoonosis [63]. So far, HEV genotypes of the *Orthohepevirus A* species have been associated with zoonotic potential. There are several sources that highlighted the zoonotic potential of rodent-borne HEV. Zoonotic potential was illustrated by a rat HEV isolate that induced a persistent infection in a patient with a liver transplant in China [64]. Additionally, there is serological evidence of rat HEV infection in German forestry workers [65], as well as in hospitalized patients with febrile illness in Vietnam [66]. There are reports that link rat HEV to severe acute hepatitis in immunocompetent patients in Canada [67].

The reservoirs of rat-associated HEV are invasive *Rattus* species such as *R. norvegicus* and *R. rattus* [68], which, analogous to red foxes, globally expand to new (sub-) urban areas and thereby provide the appropriate environment for the transmission of wildlife-associated HEV strains to the human population. Several studies confirmed a higher anti-HEV prevalence in persons with occupational contact with wild animals (hunters, foresters, veterinarians, forestry workers) when compared to the general population [65,69]. In forest workers, an anti-HEV antibody prevalence of 18% in Germany and 36% in France was reported [65].

Available data on the distribution of jackals and their role as potential reservoirs of zoonotic pathogens are scarce. It is believed that jackals migrate from southern Europe, e.g., Croatia or southern Hungary, to northern latitudes, e.g., Austria, Norway, and the Netherlands [70,71], due to climate changes. Since the jackal population is expanding rapidly, they are in competition with red fox populations [71,72]. Consequently, foxes are moving to new areas, thus contributing to the spread of fox-borne infections. Thus, jackals seem to have an increasing impact on the spread of diseases, especially since it is known that jackals harbor *Ehrlichia canis*, *Leishmania donovani*, *Toxoplasma gondii*, *Ancylostoma caninum*, *Echinococcus granulosus*, and several canine viruses [73]. A study conducted in a neighboring country, Serbia, reported the infection of the jackal population with the important zoonotic agents *Leishmania* species and *Brucella canis* [74]. The importance of jackals in the epidemiology of zoonotic diseases in newly occupied territories has been poorly investigated.

The estimated number of red foxes in Croatia, according to the data from the Ministry of Agriculture, is 15,000. Red foxes feed mostly on small rodents, but also on rabbits, which are susceptible to HEV [75,76]. Since the red fox is entering the outskirts of cities in search of food, possible direct or indirect contact with people cannot be excluded and must be monitored. The estimated jackal population size in Croatia is approximately 10,000; however, their number and the localities they inhabit are rapidly increasing. Moreover, as members of the *Canidae* family, the red fox and jackal have been previously found to be carriers of a number of viruses with zoonotic potential [24,25,26,27,77,78]. The detection of a naturally infected forest mouse as a competent host of an HEV-3 genotype that has been found to be genetically identical to previously detected strains derived from humans, wild boars, and swine in Croatia [24] may represent an epidemiological interspecies “link” between wildlife and domestic animals, and have an important role in maintaining the virus in the environment, considering the fact that red foxes and jackals feed mostly on small rodents.

The surveillance of HEV is very important worldwide in order to decrease the knowledge gap in terms of its transmission and reservoirs, considering its zoonotic potential, so this study was conducted to investigate the presence of HEV in red foxes and jackals living in natural conditions in the Republic of Croatia. More studies are needed to investigate the serological and virological prevalence, and genomic diversity of fox-derived HEV samples, as well as traditional diet composition analysis [79] of infected animals in order to understand HEV infection patterns and transmission routes at the wildlife–livestock–humans interface.

## 5. Conclusions

Although the circulation of HEV genotypes of the *Orthohepevirus A* species was not detected in the tested red fox and jackal samples during the study period, further studies should be conducted to monitor possible fluctuations in the HEV epidemiology with the consequent risk of transmission of HEV to humans and other wild mammals. Additionally, an HEV survey using RT-PCR protocols specific to HEV strains within the *Orthohepevirus C* species should be conducted on red fox and jackal samples. Future studies should be dedicated to comparing the diagnostic performance of the multispecies ELISA kit used with the HEV Wantai anti-HEV IgM assay, which is described as more sensitive.

## Figures and Tables

**Table 1:** Number of feces and muscle extract samples tested per county.

County	Number of Feces Samples of Red Foxes	Number of Muscle Samples of Red Foxes	Number of Feces Samples of Jackals	Number of Muscle Samples of Jackals
County of Zagreb	28	33	5	3
County of Krapina-Zagorje	16	15	0	0
County of Sisak-Moslavina	28	44	12	12
County of Karlovac	37	40	18	2
County of Varaždin	25	7	0	0
County of Koprivnica-Križevci	0	13	0	0
County of Bjelovar-Bilogora	2	25	0	0
County of Primorje-Gorski Kotar	27	48	9	0
County of Lika-Senj	6	49	0	2
County of Virovitica-Podravina	5	9	8	0
County of Požega-Slavonija	8	11	1	0
County of Slavonski Brod-Posavina	7	15	15	6
County of Zadar	0	3	0	1
County of Osijek-Baranja	51	28	27	10
County of Šibenik-Knin	0	18	0	2
County of Vukovar-Srijem	31	9	26	3
County of Split-Dalmacija	0	0	0	0
County of Istria	24	14	7	2
County of Dubrovnik-Neretva	0	0	0	0
County of Međimurje	7	2	0	0
City of Zagreb	7	0	0	0
	309	383	128	43
Σ	692		171	

## Data Availability

The datasets used and/or analyzed within the frame of the present study can be provided by the corresponding author upon a justified request.

## References

[1] World Health Organization Hepatitis E. https://www.who.int/news-room/fact-sheets/detail/hepatitis-e.

[2] Khuroo M.S. (2011). Discovery of hepatitis E: The epidemic non-A, non-B hepatitis 30 years down the memory lane. Virus Res..

[3] Emerson S.U., Purcell R.H. (2002). Hepatitis E virus. Rev. Med. Virol..

[4] Okamoto H. (2007). Genetic variability and evolution of hepatitis E virus. Virus Res..

[5] Tam A.W., Smith M.M., Guerra M.E., Huang C.C., Bradley D.W., Fry K.E., Reyes G.R. (1991). Hepatitis E virus (HEV): Molecular cloning and sequencing of the full-length viral genome. Virology.

[6] LeDesma R., Nimgaonkar I., Ploss A. (2019). Hepatitis E virus replication. Viruses.

[7] International Committee on Taxonomy of Viruses: ICTV. https://ictv.global/taxonomy.

[8] Purdy M.A., Harrison T.J., Jameel S., Meng X.-J., Okamoto H., Van Der Poel W.H.M., Smith D.B. (2017). ICTV Report Consortium ICTV Virus Taxonomy Profile: Hepeviridae. J. Gen. Virol..

[9] Takova K., Koynarski T., Minkov I., Ivanova Z., Toneva V., Zahmanova G. (2020). Increasing Hepatitis E Virus Seroprevalence in Domestic Pigs and Wild Boar in Bulgaria. Animals.

[10] Spahr C., Knauf-Witzens T., Vahlenkamp T., Ulrich R.G., Johne R. (2017). Hepatitis E virus and related viruses in wild, domestic and zoo animals: A review. Zoonoses Public Health.

[11] Ryll R., Heckel G., Corman V.M., Drexler J.F., Ulrich R.G. (2019). Genomic and spatial variability of a European common vole hepevirus. Arch. Virol..

[12] Kenney S.P., Meng X.-J. (2018). Hepatitis E Virus Genome Structure and Replication Strategy. Cold Spring Harb. Perspect. Med..

[13] Van der Poel W.H. (2014). Food and environmental routes of hepatitis E virus transmission. Curr. Opin. Virol..

[14] Yugo D.M., Meng X.-J. (2013). Hepatitis E virus: Foodborne, waterborne and zoonotic transmission. Int. J. Environ. Res. Public Health.

[15] Andraud M., Dumarest M., Cariolet R., Aylaj B., Barnaud E., Eono F., Pavio N., Rose N. (2013). Direct contact and environmental contaminations are responsible for HEV transmission in pigs. Vet. Res..

[16] Kasorndorkbua C., Guenette D.K., Huang F.F., Thomas P.J., Meng X.-J., Halbur P.G. (2004). Routes of transmission of swine hepatitis E virus in pigs. J. Clin. Microbiol..

[17] Said B., Ijaz S., Kafatos G., Booth L., Thomas H.L., Walsh A., Ramsay M., Morgan D. (2009). Hepatitis E outbreak on cruise ship. Emerg. Infect. Dis..

[18] Tamada Y., Yano K., Yatsuhashi H., Inoue O., Mawatari F., Ishibashi H. (2004). Consumption of wild boar linked to cases of hepatitis E. J. Hepatol..

[19] Tei S., Kitajima N., Takahashi K., Mishiro S. (2003). Zoonotic transmission of hepatitis E virus from deer to human beings. Lancet.

[20] Yazaki Y., Mizuo H., Takahashi M., Nishizawa T., Sasaki N., Gotanda Y., Okamoto H. (2003). Sporadic acute or fulminant hepatitis E in Hokkaido, Japan, may be food-borne, as suggested by the presence of hepatitis E virus in pig liver as food. J. Gen. Virol..

[21] Krumbholz A., Mohn U., Lange J., Motz M., Wenzel J.J., Jilg W., Walther M., Straube E., Wutzler P., Zell R. (2012). Prevalence of hepatitis E virus-specific antibodies in humans with occupational exposure to pigs. Med. Microbiol. Immunol..

[22] Raj V.S., Smits S.L., Pas S.D., Provacia L.B., Moorman-Roest H., Osterhaus A., Haagmans B.L. (2012). Novel Hepatitis E Virus in Ferrets, the Netherlands. Emerg. Infect. Dis..

[23] Krog J.S., Breum S.Ø., Jensen T.H., Larsen L.E. (2013). Hepatitis E Virus Variant in Farmed Mink, Denmark. Emerg. Infect. Dis..

[24] Johnson N., Freuling C., Vos A., Un H., Valtchovski R., Turcitu M., Dumistrescu F., Vuta V., Velic R., Sandrac V. (2008). Epidemiology of rabies in Southeast Europe. Dev. Biol..

[25] Bodewes R., Ruiz-Gonzalez A., Schapendonk C.M., Brand J.M.A.V.D., Osterhaus A., Smits S.L. (2014). Viral metagenomic analysis of feces of wild small carnivores. Virol. J..

[26] Szewczyk T., Werszko J., Myczka A.W., Laskowski Z., Karbowiak G. (2019). Molecular detection of *Anaplasma phagocytophilum* in wild carnivores in north-eastern Poland. Parasites Vectors.

[27] Takumi K., Sprong H., Hofmeester T.R. (2019). Impact of vertebrate communities on Ixodes ricinus-borne disease risk in forest areas. Parasites Vectors.

[28] Bodewes R., Van Der Giessen J., Haagmans B.L., Osterhaus A., Smits S.L. (2013). Identification of Multiple Novel Viruses, Including a Parvovirus and a Hepevirus, in Feces of Red Foxes. J. Virol..

[29] Eiden M., Dähnert L., Spoerel S., Vina-Rodriguez A., Schröder R., Conraths F.J., Groschup M.H. (2020). Spatial-Temporal Dynamics of Hepatitis E Virus Infection in Foxes (*Vulpes vulpes*) in Federal State of Brandenburg, Germany, 1993–2012. Front. Microbiol..

[30] Lanszki Z., Kurucz K., Zeghbib S., Kemenesi G., Lanszki J., Jakab F. (2020). Identification of Hepatitis E Virus in the Feces of Red Foxes (*Vulpes vulpes*). Animals.

[31] Lanszki J., Heltai M., Kövér G., Zalewski A. (2019). Non-linear relationship between body size of terrestrial carnivores and their trophic niche breadth and overlap. Basic Appl. Ecol..

[32] Soe E., Davison J., Süld K., Valdmann H., Laurimaa L., Saarma U. (2017). Europe-wide biogeographical patterns in the diet of an ecologically and epidemiologically important mesopredator, the red fox Vulpes vulpes: A quantitative review. Mammal Rev..

[33] Johne R., Dremsek P., Reetz J., Heckel G., Hess M., Ulrich R.G. (2014). Hepeviridae: An expanding family of vertebrate viruses. Infect. Genet. Evol..

[34] Xia J., Zeng H., Liu L., Zhang Y., Liu P., Geng J., Wang L., Wang L., Zhuang H. (2015). Swine and rabbits are the main reservoirs of hepatitis E virus in China: Detection of HEV RNA in feces of farmed and wild animals. Arch. Virol..

[35] Jemeršić L., Roić B., Balatinec J., Keros T. (2010). Hepatitis E—Are we threatened? Hepatitis E—Jesmo li ugroženi?. Vet. Stanica.

[36] Prpić J., Černi S., Škorić D., Keros T., Brnić D., Cvetnić Ž., Jemeršić L. (2015). Distribution and molecular characterization of Hepatitis E virus in domestic animals and wildlife in Croatia. Food Environ. Virol..

[37] Jemeršić L., Keros T., Maltar L., Barbić L., Vilibić-Cavlek T., Jeličić P., Ðaković-Rode O., Prpić J. (2017). Differences in hepatitis E virus (HEV) presence in naturally infected seropositive domestic pigs and wild boars—An indication of wild boars having an important role in HEV epidemiology. Vet. Arh..

[38] Jemeršić L., Prpić J., Brnić D., Keros T., Pandak N., Đaković-Rode O. (2019). Genetic diversity of hepatitis E virus (HEV) strains derived from humans, swine and wild boars in Croatia from 2010 to 2017. BMC Infect. Dis..

[39] Burri C., VIal F., Ryser-Degiorgis M.P., Schwermer H., Darling K., Reist M., Wu N., Beerli O., Schoning J., Cavassini M. (2014). Seroprevalence of hepatitis E virus in domestic pigs and wild boars in Switzerland. Zoonoses Public Health.

[40] Anita A., Gorgan L., Anita D., Oslobanu L., Pavio N., Savuta G. (2014). Evidence of hepatitis E infection in swine and humans in East Region of Romania. Int. J. Infect. Dis..

[41] Montagnaro S., De Martinis C., Sasso S., Ciarci R., Damiano S., Auletta L., Iovane V., Zottola T., Pagnini U. (2015). Viral and antibody prevalence of hepatitis E in European wild boars (*Sus scrofa*) and hunters at Zoonotic risk in the Latium Region. J. Comp. Pathol..

[42] Thiry D., Mauroy A., Saegerman C., Licoppe A., Fett T., Thomas I., Brochier B., Thiry E., Linden A. (2015). Belgian wildlife as potential zoonotic reservoir of hepatitis E. Trans. Emerg. Dis..

[43] Mazzei M., Forzan M., Pizzurro F., Picciolli F., Bandecchi P., Poli A. (2015). Detection of hepatitis E virus antibodies in domestic and wild animal species in central Italy. Clin. Microbiol..

[44] Prpić J., Keros T., Vucelja M., Bjedov L., Ðaković-Rode O., Margaletić J., Habrun B., Jemeršić L. (2019). First evidence of hepatitis E virus infection in a small mammal (yellow-necked mouse) from Croatia. PLoS ONE.

[45] Jothikumar N., Cromeans T.L., Robertson B.H., Meng X.J., Hill V.R. (2006). A broadly reactive one-step real-time RT-PCR assay for rapid and sensitive detection of hepatitis E virus. J. Virol. Methods.

[46] Kruse H. (2004). Wildlife as source of zoonotic infections. Emerg. Infect. Dis. J..

[47] World Health Organization WHO Consultation on Public Health and Animal Transmissible Spongiform Encephalopathies: Epidemiology, Risk and Research Requirements, Geneva, Switzerland, 1–3 December 1999. https://apps.who.int/iris/handle/10665/66422.

[48] Weiss S., Nowak K., Fahr J., Wibbelt G., Mombouli J.V., Parra H.J., Leendertz F. (2012). Henipavirus-related sequences in fruit bat bushmeat, Republic of Congo. Emerg. Infect. Dis..

[49] Pernet O., Schneider B.S., Beaty S.M., LeBreton M., Yun T.E., Park A., Zachariah T.T., Bowden T.A., Hitchens P., Ramirez C.M. (2014). Evidence for henipavirus spillover into human populations in Africa. Nat. Commun..

[50] Taylor L.H., Latham S.M., Mark E. (2001). Risk factors for human disease emergence. Philos. Trans. R. Soc. Lond. Ser. B. Biol. Sci..

[51] Williams E., Yuill T., Artois M., Fischer J., Haigh S. (2002). Emerging infectious diseases in wildlife. Rev. Sci. Tech. Off. Int. Epizoot..

[52] Johnson C.K., Hitchens P.L., Pandit P.S., Rushmore J., Evans T., Smiley Y., Cristine C.W., Doyle M.M. (2020). Global shifts in mammalian population trends reveal key predictors of virus spillover risk. Proc. R. Soc. B..

[53] Wolfe N.D., Daszak P., Kilpatrick A.M., Burke D.S. (2005). Bushmeat, hunting, deforestation, and prediction of zoonotic disease emergence. Emerg. Infect. Dis..

[54] Johne R., Plenge-Bönig A., Hess M., Ulrich R.G., Reetz J., Schielke A. (2009). Detection of a novel hepatitis E-like virus in faeces of wild rats using a nested broad-spectrum RT-PCR. J. Gen. Virol..

[55] Bedeković T., Lemo N., Lojkić I., Mihaljević Z., Jungić A., Cvetnić Z., Cač Z., Hostnik P. (2013). Modification of the fluorescent antibody virus neutralisation test—Elimination of the cytotoxic effect for the detection of rabies virus neutralising antibodies. J. Virol. Methods.

[56] Wen G.P., Tang Z.M., Yang F., Zhang K., Ji W.F., Cai W., Huang S.J., Wu T., Zhang J., Zheng Z.Z. (2015). A valuable antigen detection method for diagnosis of acute hepatitis E. J. Clin. Microbiol..

[57] Zhao C., Geng Y., Harrison T.J., Huang W., Song A., Wang Y. (2015). Evaluation of an antigen-capture EIA for the diagnosis of hepatitis E virus infection. J. Viral Hepat..

[58] Lu J., Huang Y., Wang P., Li Q., Li Z., Jiang J., Guo Q., Gui H., Xie Q. (2021). Dynamics of Hepatitis E Virus (HEV) Antibodies and Development of a Multifactorial Model To Improve the Diagnosis of HEV Infection in Resource-Limited Settings. J. Clin. Microbiol..

[59] Haydon D.T., Cleaveland S., Taylor L.H., Laurenson M.K. (2002). Identifying reservoirs of infection: A conceptual and practical challenge. Emerg. Infect. Dis..

[60] Hassell J.M., Begon M., Ward M.J., Fèvre E.M. (2017). Urbanization and disease emergence: Dynamics at the wildlife-livestock-human Interface. Trends Ecol. Evol..

[61] Mackenstedt U., Jenkins D., Romig T. (2015). The role of wildlife in the transmission of parasitic zoonoses in peri-urban and urban areas. Int. J. Parasitol. Parasites Wildl..

[62] Najm N.A., Meyer-Kayser E., Hoffmann L., Herb I., Fensterer V., Pfister K., Silaghi C. (2014). A molecular survey of *Babesia* spp. and *Theileria* spp. in red foxes (*Vulpes vulpes*) and their ticks from Thuringia, Germany. Ticks Tick Borne Dis..

[63] Vuitton D.A., Demonmerot F., Knapp J., Richou C., Grenouillet F., Chauchet A., Vuitton L., Bresson-Hadni S., Millon L. (2015). Clinical epidemiology of human AE in Europe. Vet. Parasitol..

[64] Sridhar S., Yip C.C., Wu S., Cai J., Zhang A.J.-X., Leung K.-H., Chung T.W., Chan J.F., Chan W.-M., Teng J.L. (2018). Rat Hepatitis E Virus as Cause of Persistent Hepatitis after Liver Transplant. Emerg. Infect. Dis..

[65] Dremsek P., Wenzel J.J., Johne R., Ziller M., Hofmann J., Groschup M.H., Werdermann S., Mohn U., Dorn S., Motz M. (2011). Seroprevalence study in forestry workers from eastern Germany using novel genotype 3- and rat hepatitis E virus-specific immunoglobulin G ELISAs. Med. Microbiol. Immunol..

[66] Shimizu K., Hamaguchi S., Ngo C.C., Li T.-C., Ando S., Yoshimatsu K., Yasuda S.P., Koma T., Isozumi R., Tsuda Y. (2016). Serological evidence of infection with rodent-borne hepatitis E virus HEV-C1 or antigenically related virus in humans. J. Vet. Med. Sci..

[67] Andonov A., Robbins M., Borlang J., Cao J., Hatchette T., Stueck A., Deschambault Y., Murnaghan K., Varga J., Johnston L. (2019). Rat Hepatitis E Virus Linked to Severe Acute Hepatitis in an Immunocompetent Patient. J. Infect. Dis..

[68] Ryll R., Eiden M., Heuser E., Weinhardt M., Ziege M., Höper H., Groschup M.H., Heckel G., Johne R., Ulrich R.G. (2018). Hepatitis E virus in feral rabbits along a rural-urban transect in Central Germany. Infect. Genet. Evol..

[69] Chaussade H., Rigaud E., Allix A., Carpentier A., Touzé A., Delzescaux D., Choutet P., Garcia-Bonnet N., Coursaget P. (2013). Hepatitis E virus seroprevalence and risk factors for individuals in working contact with animals. J. Clin. Virol..

[70] Arnold J., Humer A., Heltai M., Murariu D., Spassov N., Hackländer K. (2012). Current status and distribution of golden jackals *Canis aureus* in Europe. Mammal Rev..

[71] Duscher G.G., Kübber-Heiss A., Richter B., Suchentrunk F. (2013). A golden jackal (*Canis aureus*) from Austria bearing Hepatozoon canis—Import due to immigration into a non-endemic area?. Ticks Tick Borne Dis..

[72] Majláthová V., Hurníková Z., Majláth I., Petko B. (2007). *Hepatozoon canis* infection in Slovakia: Imported or autochthonous?. Vector Borne Zoonotic Dis..

[73] Shamir M., Yakobson B., Baneth G., King R., Dar-Verker S., Markovics A., Aroch I. (2001). Antibodies to selected canine pathogens and infestation with intestinal helminths in golden jackals (*Canis aureus*) in Israel. Vet. J..

[74] Cirović D., Chochlakis D., Tomanović S., Sukara R., Penezić A., Tselentis Y., Psaroulaki A. (2014). Presence of *Leishmania* and *Brucella* species in the golden jackal *Canis aureus* in Serbia. BioMed Res. Int..

[75] Zhang Y., Gong W., Song W.T., Fu H., Wang L., Li M., Wang L., Zhuang H. (2017). Different susceptibility and pathogenesis of rabbit genotype 3 hepatitis E virus (HEV-3) and human HEV-3 (JRC-HE3) in SPF rabbits. Vet. Microbiol..

[76] Li S., He Q., Yan L., Li M., Liang Z., Shu J., Zhang F., Wang L., Wang L. (2020). Infectivity and pathogenicity of different hepatitis E virus genotypes/subtypes in rabbit model. Emerg. Microbes Infect..

[77] Davidson W.R., Appel M.J., Doster G.L., Baker O.E., Brown J.F. (1992). Diseases and parasites of red foxes, gray foxes, and coyotes from commercial sources selling to fox-chasing enclosures. J. Wildl. Dis..

[78] Goller K.V., Fickel J., Hofer H., Beier S., East M.L. (2013). Coronavirus genotype diversity and prevalence of infection in wild carnivores in the Serengeti National Park, Tanzania. Arch. Virol..

[79] Lanszki Z., Horváth G.F., Bende Z., Lanszki J. (2019). Differences in the diet and trophic niche of three sympatric carnivores in a marshland. Mammal Res..

[80] Jemeršić L., Lojkić I., Krešić N., Keros T., Zelenika T.A., Jurinović L., Skok D., Bata I., Boras J., Habrun B. (2021). Investigating the Presence of SARS CoV-2 in Free-Living and Captive Animals. Pathogens.

